# Breast Gangrene Associated With Thrombotic Vasculopathy: A Case Report

**DOI:** 10.7759/cureus.103087

**Published:** 2026-02-06

**Authors:** Erika Judith Damián-Magaña, Abraham Isaí Cabello-Hernández, Sabrina Escandón-Pérez, Luisa Mariana Guerrero-Escudero, Cristina Berumen-Glinz, Sonia Toussaint-Caire

**Affiliations:** 1 Department of Dermatology, Hospital General Dr. Manuel Gea Gonzalez, Mexico City, MEX; 2 Department of Dermatopathology, Hospital General Dr. Manuel Gea Gonzalez, Mexico City, MEX

**Keywords:** breast gangrene, rheumatoid arthritis, thrombosis, thrombotic vasculopathy, venous thrombosis

## Abstract

This article aimed to report a patient with breast gangrene secondary to occlusive thrombotic vasculopathy. A 50-year-old woman with a history of long-standing rheumatoid arthritis presented with a rapidly progressing necrotic ulcer on her right breast. Tests showed active rheumatoid arthritis. Biopsy revealed epidermal necrosis and thrombotic vasculopathy with fibrinous thrombi. The patient was diagnosed with breast gangrene secondary to thrombotic vasculopathy associated with rheumatoid arthritis. Prednisone and methotrexate were initiated with a favorable response, but the patient was subsequently lost to follow-up. Breast gangrene is a rare and potentially life-threatening condition, most commonly associated with necrotizing fasciitis and favored by severe infections, microangiopathy, multiple comorbidities, trauma, iatrogenic procedures, immunosuppression, and idiopathic forms. Management relies on prompt surgical debridement and broad-spectrum antibiotic therapy; in extensive cases, reconstructive surgery or mastectomy may be required due to the condition's high morbidity and mortality. Although it has been linked to other autoimmune diseases, an association with thrombotic vasculopathy has not been previously reported.

## Introduction

Breast gangrene is a rare condition that is either idiopathic or secondary to another triggering factor, such as necrotizing fasciitis, invasive procedures, or drugs, among others. Clinically, it typically begins as unilateral mastitis and rapidly progresses to dermal gangrene with eschar formation; in rare cases, it may extend to deeper tissues as necrotizing fasciitis [1]. Standard management includes debridement and antibiotic treatment. Histopathological examination reveals acute inflammatory infiltrate, severe necrosis, necrotizing arteritis, and venous thrombosis [1]. We present a case of breast gangrene associated with thrombotic vasculopathy.

## Case presentation

**Figure 1:**
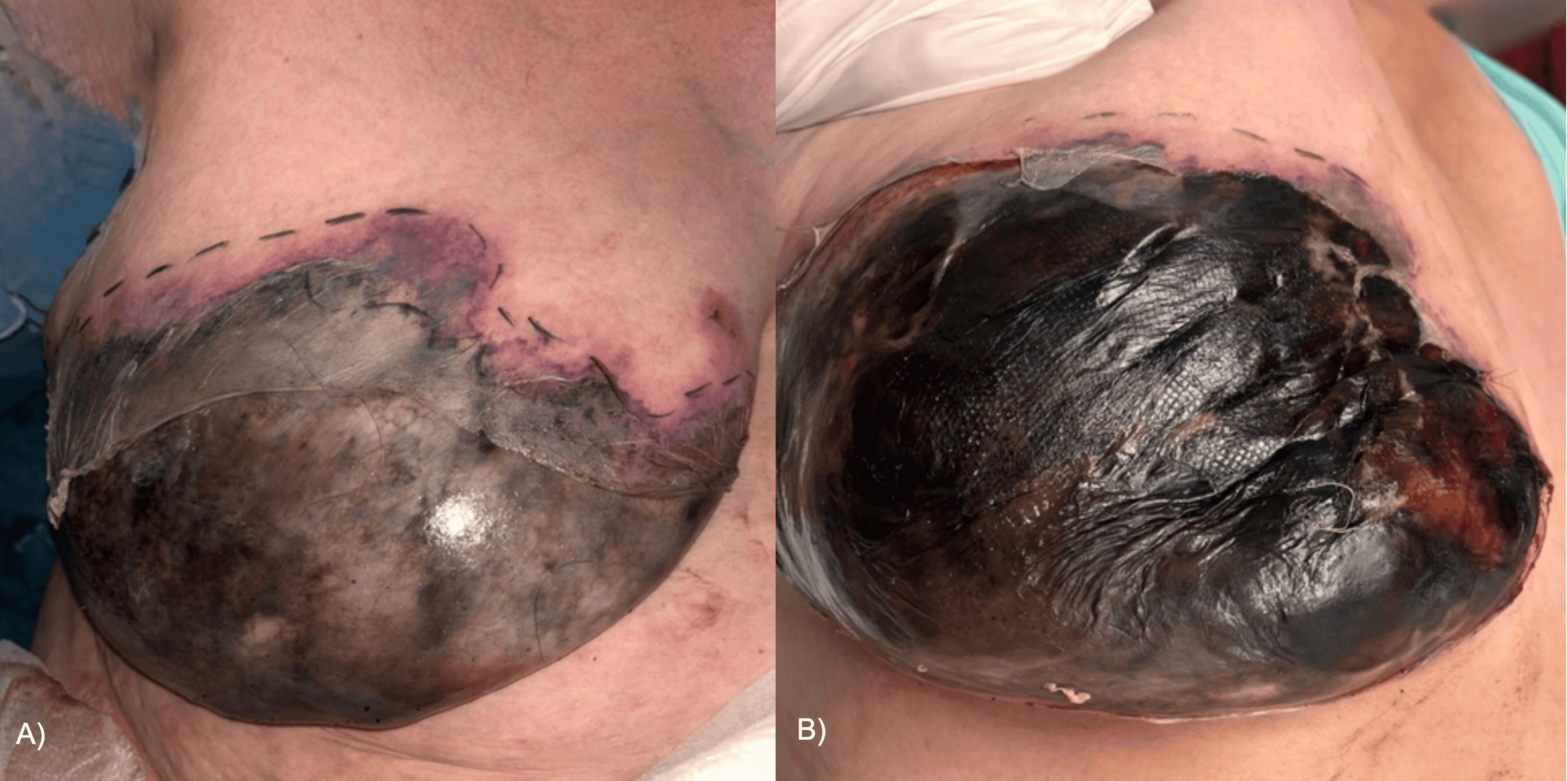
Clinical image of the right breast. (A) Initial presentation. (B) Right breast 24 hours later.

A 50-year-old woman presented to our service with a three-day history of erythema and induration of the right breast. She had a history of type 2 diabetes and systemic hypertension, both under adequate control, and rheumatoid arthritis, diagnosed 20 years ago, treated with prednisone. On examination, she presented a dermatosis localized on the right breast characterized by a 15 × 20 cm irregular ulcer with necrotic tissue and eschar covering approximately 70% of the breast, accompanied by areas of epidermal denudation and peripheral erythema (Figure 1). The patient reported moderate pain. Vital signs were within normal limits (temperature: 97.16 °F, respiratory rate: 18 bpm, heart rate: 65 bpm, blood pressure: 127/75 mmHg). Differential diagnoses, such as necrotizing fasciitis, pyoderma gangrenosum, warfarin necrosis, calciphylaxis, rheumatoid vasculitis, and inflammatory breast cancer, were considered.

**Figure 2:**
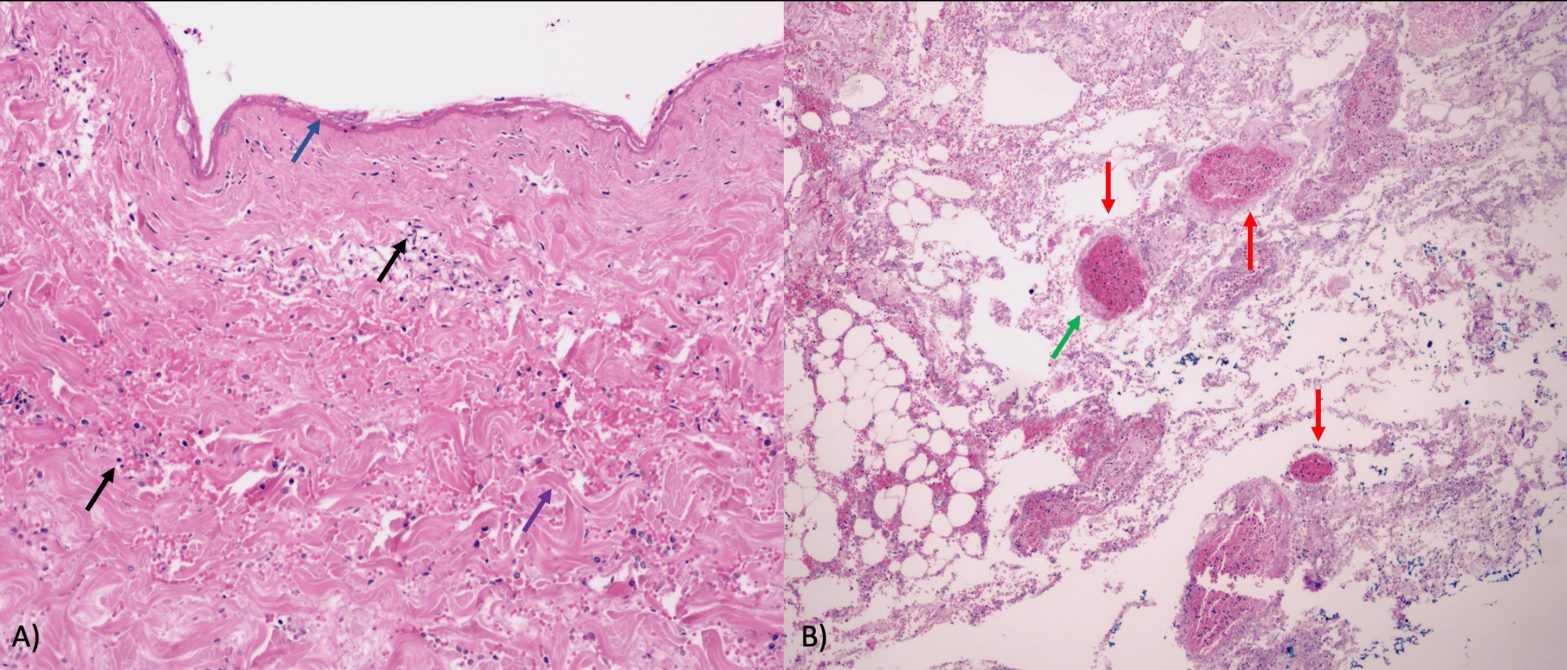
Histological image of the right breast skin. (A) Full-thickness epidermal necrosis (blue arrow); superficial and deep perivascular lymphocytic infiltrate (black arrows); thickened collagen fibers (purple arrow). (B) Fibrous thrombi within the vascular lumen (red arrows); vascular wall edema (green arrow, hematoxylin-eosin stain; original magnification ×10).

Hospital admission was decided upon after treatment. Laboratory tests revealed increased leukocytes of 12 cells/µL (normal limits: 4.5-11 cells/µL), neutrophils of 11.06 cells/µL (normal limits: 1.5-8 cells/µL), glucose of 476 mg/dL (normal limits: <100 md/dL), and C‑reactive protein (CRP) of 53.7 md/dL (normal limits: <1 mg/dL). Antibody titers were low (lupus anticoagulant: 22 U/mL, anticardiolipin: 15 U/mL, anti-β2-glycoprotein I: 12 U/mL), which ruled out the presence of antiphospholipid syndrome. We performed a chest X-ray, but it did not show any significant abnormalities. Subsequently, a skin biopsy was performed for histopathological study, which revealed necrosis throughout the thickness of the epidermis. In the dermis, there was a notable presence of superficial and deep perivascular infiltrate composed of lymphocytes with thickened collagen fibers. In the deep dermis, blood vessels with edema in the wall and fibrinous thrombi in the wall of some vessels were observed. Periodic acid-Schiff (PAS) staining highlighted the presence of fibrotic thrombi and Gram-negative bacteria without the presence of microorganisms (Figure 2). Vasculitis was excluded due to the absence of fibrinoid necrosis, leukocytoclasia, or vessel wall destruction. 

The patient completed a 14-day course of intravenous penicillin for *Klebsiella pneumoniae*, initiated after its empirical isolation from a secretion culture. However, no microorganisms were identified on quantitative tissue Gram staining and culture; therefore, this finding was interpreted as colonization, and the diagnosis of necrotizing fasciitis was excluded. Following the above approach, a diagnosis of breast gangrene associated with thrombotic vasculopathy was made. The patient underwent surgical debridement and was subsequently enrolled in a breast reconstruction protocol; however, she was lost to follow-up. 

## Discussion

Breast gangrene is a rare condition with few cases reported in the literature. Breast gangrene has been associated with necrotizing fasciitis, which is why it has been considered a type of Fournier's gangrene favored by a massive and complicated infection with a picture of obliterative arteritis [1]. The type of necrosis in breast gangrene is coagulative necrosis or dry necrosis. Most reported cases have also been associated with conditions such as mastitis, breast abscesses, and consumption of anticoagulants such as warfarin [2]. Other predisposing factors described include metastasis from solid organ neoplasia, pregnancy, breastfeeding, diabetes, trauma, immunosuppression, thrombophlebitis, peripheral arterial disease, carbon monoxide poisoning, breast cancer, radiotherapy, cardiac revascularization surgery, complications from puerperal sepsis, and even idiopathic cases [3,4]. Anecdotally, its appearance has been reported after the injection of methylene blue for sentinel lymph node biopsy and after breast biopsy [5,6]. In patients with previously irradiated breast cancer and diabetic microvascular disease, previous minor trauma can cause localized breast gangrene and slowly progress to affect the entire breast [7]. In patients with a history of diabetes, the predisposition to this condition could be explained by hyperglycemia and the coexistence of atherosclerosis. A spontaneous presentation of unknown etiology was first reported by Cutter in 1924 as a case of breast apoplexy [8]. Despite the associations described, the nature and etiopathogenesis of this entity remain poorly described.

A retrospective study evaluating 10 patients with breast gangrene found that the main causes were belladonna application (4/10), *S. aureus *infection secondary to bites (3/10), and iatrogenic trauma due to aspiration of an erythematous area of the breast under septic conditions (1/10). In this study, 90% of cases required surgical debridement [1]. Recently, its appearance has been reported in a case of HIV infection [9]. On the other hand, the presence of breast gangrene has been described in a case of COVID-19 infection, probably favored by vascular insufficiency aggravated by a secondary infection [7].

In idiopathic forms, breast pain occurs without a history of infection or trauma, and an area of orange-peel skin initially develops [1]. Clinically, it begins as a usually unilateral case of mastitis, which progresses within two to five days to dermal gangrene, leading to the formation of eschar [1]. In exceptional cases, it can involve deeper tissues and present as necrotizing fasciitis. Cases precipitated by other conditions vary in terms of progression time and accompanying symptoms.

During physical examination, the presence of subcutaneous emphysema should be ruled out, and the approach should be complemented with computed axial tomography to search for collections and assess the condition of the fascia and pectoral muscles to rule out gas gangrene, which is considered a surgical emergency [10].

Breast gangrene is recognized as a serious complication in patients with diabetes; however, other factors, such as atherosclerosis and hyperglycemia, contribute to susceptibility to gangrene [1]. Regarding its association with autoimmune comorbidities, breast ulcers secondary to cutaneous polyarteritis nodosa have been reported, although their occurrence in rheumatoid arthritis has not been reported [11]. The prothrombotic state in rheumatoid arthritis may be precipitated by multiple factors, including persistent systemic inflammation, which promotes endothelial injury, platelet activation, and increased expression of procoagulant factors [12]. Despite the associations mentioned, no relationship has been established with thrombotic processes, as in our case; however, it is necessary to rule out rheumatoid vasculitis as a significant trigger in a patient with rheumatoid arthritis.

Microbiological studies reveal aerobic flora, notably *S. aureus*, *Escherichia coli*, *Proteus*, and *Enterococcus*, among others [13]. Venous occlusions with microthrombi in histopathological studies have been reported in cases associated with panarteritis, focal obliterative endarteritis, and small vessel inflammation. However, in our case, the presence of vasculitis has been ruled out through histopathological study. It is considered a potentially fatal condition that can lead to severe sepsis, which confers a mortality rate of 20-70% [14].

Therapeutic management requires a multidisciplinary approach involving surgical intervention, aggressive debridement of necrotic tissue (as in our case), administration of broad-spectrum antibiotics, and the use of aseptic dressings. Serial debridement is required in cases where a large area of the body is affected. Once viable tissue is present, the use of grafts is an option. In cases of extensive involvement, mastectomy is even considered, after which primary closure may not be advisable. In our case, the loss of follow-up prevented us from offering complete surgical management and better aesthetic results, which represents a limitation of this case.

## Conclusions

Breast gangrene is a rare entity with a multifactorial etiology, making it imperative to rule out infectious causes. While it has been described in association with other autoimmune conditions, this case is a rare example of breast gangrene associated with thrombotic vasculopathy. In patients with necrotic breast ulcers and underlying autoimmune disease, thrombotic vasculopathy should be considered, even when there is no evidence of overt infection or vasculitis. In the absence of clinical or laboratory evidence of infection, histopathological examination is essential for determining the etiology.
